# Australian Dog Owners’ Acceptance of Insect-Based Pet Food

**DOI:** 10.3390/insects16030290

**Published:** 2025-03-11

**Authors:** Anna Triggs, Ishka Bless, Lukas Danner, Maria Saarela, Kerry Wilkinson

**Affiliations:** 1 School of Agriculture, Food and Wine, The University of Adelaide, PMB 1, Glen Osmond, SA 5064, Australia; annatriggs1@gmail.com (A.T.); ishka.bless@adelaide.edu.au (I.B.); lukas.danner@csiro.au (L.D.); 2 Commonwealth Scientific and Industrial Research Organisation (CSIRO), Agriculture and Food, 671 Sneydes Rd, Werribee, VIC 3030, Australia; 3 South Australian Research and Development Institute (SARDI), Department of Primary Industries and Regions, GPO Box 397, Adelaide, SA 5001, Australia; maria.saarela@sa.gov.au

**Keywords:** black soldier fly larvae, crickets, dog-human relationship, entomophagy, food neophobia, marketing, mealworms, nutrition

## Abstract

Insect-based dog food is increasingly becoming available in pet food markets, potentially offering environmental and nutritional benefits, depending on insect species and how they are reared, processed, and utilized. However, there is limited understanding of dog owners’ attitudes towards the use of insects as protein alternatives for dogs. An online survey was conducted to explore Australian dog owners’ (*n* = 201) attitudes towards insect-based pet food, segmenting participants according to their food neophobia, previous insect consumption, and dog-human relationship. Participants’ dog food purchases were highly influenced by perceived health/nutrition, palatability, and product quality, with these factors being of most importance to ‘dog people’ (i.e., participants with closer relationships with their dogs). Participants were reasonably accepting of insect-based pet food made from mealworms and crickets and slightly accepting of products made from black soldier fly larvae. In contrast, participants’ responses to plant-based pet foods were neutral. Food neophiles, and to a lesser extent, insect-eating consumers (i.e., those who had previously eaten insects), were found to be more willing to feed their dogs insects in comparison to non-neophiles and non-insect-eating consumers. Overall, Australian dog owners appear to be supportive of feeding their dogs diets supplemented with insect protein. Insights from this study will help inform the development and marketing of insect-based pet foods.

## Simple Summary

Insects are considered to be a nutritious and sustainable alternative to traditional protein sources. However, in Western societies, there are still significant cultural barriers hindering the use of insects as food. For many, an underlying sense of disgust discourages insect consumption. In this study, we evaluated whether Australian dog owners would be willing to feed their dogs insect-based pet food, as well as the factors underpinning their attitudes—from their prior experience of eating insects and the closeness of their relationship with their dog to the type of insect and product price, quality, and health or environmental benefits. Participants were generally more accepting of insect-based pet food than plant-based pet food and were more willing to feed insect-based pet food to their dogs than to eat insects themselves. When purchasing dog food, participants placed the greatest importance on health/nutrition, palatability, and product quality. Pet food manufacturers should, therefore, consider these factors when making and marketing insect-based pet foods.

## 1. Introduction

With our global population expected to approach 10 billion people by 2050 [1], alternative protein sources will be needed to accommodate the associated protein demand, not only for human consumption, but also for livestock [2,3,4] and the rising number of companion animals (i.e., pets, especially dogs and cats) [5,6]. Entomophagy, the practice of eating insects, occurs in over 100 countries around the world (predominantly in Africa, Asia, and South America) [2,7]. Although insect consumption is still novel in many Western societies, interest in insects as a sustainable alternative protein source has rapidly increased over the last decade [4], and there has been substantial growth in the number of companies rearing insects for use in animal feed, including pet food [4,6,7,8]. Rearing insects is generally considered more environmentally sustainable than farming conventional livestock (e.g., chickens, pigs, sheep and cattle), as insects typically have lower water, feed, and space requirements, and lower greenhouse gas emissions [2,7,9,10,11] (although this varies considerably by species, and their relative inputs and outputs). While the nutritional composition of edible insects also varies by species (as well as metamorphic stage, habitat, and diet) [12], they tend to be rich in proteins, carbohydrates, fats, minerals, and vitamins [2,12].

Considerable research has been undertaken to determine consumer acceptance of insects as human food. Currently, it is well-accepted that in Western society, significant cultural barriers exist [13,14]. For many, an underlying sense of disgust lessens the likelihood of insect consumption [7,15], but some consumer segments are more receptive to insect consumption, including food neophiles (individuals who are overtly disposed to trying novel foods) and those who have previously consumed insects [13,14,15,16,17]. Some studies have also suggested that acceptance of insects as food varies by consumer gender, ethnicity, and age [16,17,18,19]. Strategies for improving willingness to consume insects have been recommended, including the use of insect meal (insects ground into ‘flour’) vs. whole insects, to overcome aversion associated with insect visibility [19,20]. Consumer attitudes towards the use of insects as feed for livestock and farmed fish have also been studied, and acceptance is generally more favorable (than for human consumption) [21,22,23,24]. Comparatively fewer studies have investigated the acceptance of insects as food for pets [25,26,27,28,29,30,31,32].

Around the world, pet ownership has increased significantly [33], and concerns have been raised over the corresponding carbon ‘pawprint’ [5,34,35]. Sustainable pet foods are gaining popularity, including those made from alternative protein sources, such as plants (e.g., soy) [36] and insects [8]. Although still very niche, the insect-based pet food market is gradually expanding. In Australia alone, several insect-based pet food brands have now become available (including BuggyBix, Petgood, Grubbo, Planet A Pet Food, ONE, Huds and Toke, Pet Food Australia, and Billy and Margot), in addition to the ongoing trade of live insects as feed for exotic pets.

Previous studies investigating the digestibility and nutritional quality of insects for dogs found that black soldier fly larvae (BSFL) are readily digested and offer a high-quality protein source [6,8,37,38,39,40,41,42,43,44]. Indeed, insect-based pet food typically exceeds the 18 g of protein/100 g dry matter levels recommended by the European Pet Food Industry (FEDIAF) for adult dogs [45]. However, there is limited research into the long-term effects of insect-based diets on dog health [8]. Furthermore, only a few studies have investigated the acceptance of insect-based pet food by dogs and/or their owners. One study suggested that dogs were attracted to the aromas of different insect species (e.g., mealworms and crickets) [25], while another reported that US dog owners were moderately accepting of pet foods made from mealworms, ants, BSFL, and crickets [26]. A recent survey of Italian dog owners suggested that food neophobia and environmental values are important determinants of willingness to pay for insect-based pet food [28].

Various factors influence dog owners’ pet food purchasing decisions, including nutrition, palatability, and their dog-human relationship (i.e., their level of companionship with their dog) [46,47]. Increasingly, dog owners are forming stronger bonds with their dogs, with some ‘humanizing’ their dogs (i.e., anthropomorphism) and/or seeing their dogs as extensions of themselves [46,48,49]. Closer bonds have been shown to increase owners’ concern over the nutrition and palatability of pet food [46] and may help explain the increased pet food expenditure and growth observed in the premium pet food market in Australia [50]. However, as demonstrated by Fantechi and coworkers, this may also act as a barrier to the purchase of insect-based pet food [28].

Despite the growing number of insect-based pet food products available in Australia (and elsewhere), the future success of the insect-based pet food industry is uncertain due to a lack of research on pet owners’ acceptance. This study, therefore, aimed to explore Australian dog owners’ willingness to feed their dogs insect-based pet food and provide insights that could be used by the industry to develop and market pet foods made from insects.

## 2. Materials and Methods

### 2.1. Online Survey

An online survey was conducted to investigate Australian dog owners’ attitudes towards, and acceptance of, insect-based pet food. Participants (*n* = 201) were recruited via convenience sampling using a combination of social media (e.g., Facebook), e-newsletters, and flyers. Inclusion criteria required participants to be at least 18 years of age, Australian residents, and dog owners. The survey was administered via SurveyMonkey^TM^ (San Mateo, CA, USA) and took participants 10–12 min to complete. Participants who completed the survey had the opportunity to enter a draw to win one of four $25 gift vouchers. The survey comprised seven sections. The first section captured consumer demographics: sex, age, education, annual household income, and animal protein consumption. The second section included questions related to: (i) participants’ awareness and experience of entomophagy; (ii) dog food purchasing decisions; and (iii) the influence of environmental factors on owners’ and dogs’ diets. The final four sections utilized seven-point Likert scales (where 1 = ‘strongly disagree’ and 7 = ‘strongly agree’) to capture participants’ responses to: (iv) the Food Neophobia Scale (FNS), developed by Pliner and Hobden [51], comprising 10 items that explore participants’ levels of food neophobia (i.e., willingness to consume novel foods); (v) questions establishing the dog-human relationship, comprising five ‘dog-oriented self-concept’ items and six ‘anthropomorphism’ items, adapted from a previous study [48]; (vi) questions investigating determinants for personal and dogs’ diets, adapted from a previous study [48]; and (vii) questions exploring willingness of owners to feed their dogs plant-based and insect-based pet foods. For responses to the FNS, total scores were summed to establish levels of food neophobia (maximum total score of 70). Participants with scores ≤25 were identified as ‘neophiles’ (i.e., highly willing to consume novel foods), and participants with scores >25 were identified as ‘non-neophiles’ (i.e., low to moderate willingness to consume new foods). Neophobes (participants with FNS scores > 45) were not considered as a separate segment due to the small sample size (*n* = 4) and were instead classified as non-neophiles. Participants were also invited to share any additional feedback and concerns with feeding their dogs’ insect-based pet food via two open-ended final questions: What, if any, concerns do you have around feeding your dog(s) insect-based pet food?; and Do you have anything else you would like to share with us?

### 2.2. Acceptance Trial

A commercial dog treat made with BSFL was purchased from BuggyBix (Milsons Point, NSW, Australia), and an acceptance trial conducted to investigate the willingness of dogs to consume insect-based dog treats. Dog owners and their dogs (*n* = 42 and 50, respectively, i.e., some participants owned more than one dog) were recruited at public locations (e.g., dog parks located in metropolitan South Australia, Australia), again via convenience sampling. Inclusion criteria required dog owners to be at least 18 years of age and Australian residents; dogs could be of any breed, but they had to be adults without any known food allergies. Prior to the tasting, dog owners completed a questionnaire comprising questions related to: (i) demographics (sex and age); (ii) their awareness of and experience with entomophagy; (iii) their dog’s/dogs’ breed and usual diet; and (iv) the potential for environmental considerations to motivate changes in dog food choice. Dogs were then offered the insect-based treats (presented by the owner). Dogs’ responses were recorded as follows: showed no interest in the treat; smelled but did not eat the treat; smelled and partially ate the treat; or smelled and fully ate the treat. Participants who completed the trial had the opportunity to enter a draw to win one of four $25 gift vouchers.

### 2.3. Data Analysis

Participants in the online survey were segmented on the basis of food neophobia (as described in Section 2.2), previous insect consumption, and dog-human relationship. Items exploring the dog-human relationship, utilized from a previous survey [48], were analyzed by hierarchical cluster analysis (Ward’s method) using SPSS (version 28, Armonk, NY, USA). Three clusters were selected to achieve the least internal variance within clusters, resulting in clusters of similar sizes (dog-human relationship groups are defined more in Section 3.1). Other data analyses included: descriptive techniques (means and percentages) using Microsoft Excel and *t*-tests or analysis of variance (ANOVA) using GenStat (23rd Edition, VSN International, Hemel Hempstead, UK).

### 2.4. Ethical Statement

Participants gave informed consent before participating in the study, which was approved by the Human Research Ethics Committee of the University of Adelaide (H-2021-071). Ethical approval was also obtained from the University of Adelaide’s Animal Ethics Committee to permit the use of animals in the research (S-2021-054).

## 3. Results and Discussion

### 3.1. Dog Owner Demographics and Awareness and Prior Consumption of Edible Insects

Table 1 reports the demographics of the 201 participants who completed the online survey. A substantial proportion of participants were female (*n* = 165, 82.1%), which is not surprising, given that the majority (64%) of Australian pet owners are female [52]. The sample was predominantly composed of younger individuals, with nearly 50% being under 35 years of age. The largest group of participants was 18–24-year-olds (29.4%). This agrees with a reported 70% of Australians aged 18–24 owning pets [52]. The relatively high response rate from younger participants might reflect the distribution of the online survey via social media platforms. Additionally, a significant portion of participants held an undergraduate or postgraduate degree (*n* = 117, 58.2%), suggesting potential gender, age, and educational bias within the sample. Household income varied, ranging from $50,000 to $150,000 per annum for just over half of the participants (*n* = 106, 52.7%). Ten percent of participants (*n* = 20) were employed in animal-related roles (e.g., as veterinarians, animal trainers, or pet store employees), limiting any potential study bias from participants who may have better insight into animal health and nutrition than the average pet owner. Participants were asked about their animal protein consumption to identify any vegetarians or vegans, i.e., consumer segments that might be expected to be less willing to consume insects. However, only nine participants indicated that their diets did not contain any animal products; hence, dietary choice was not investigated further. Most participants had heard of entomophagy (*n* = 161, 80.1%), but only a third stated they had previously consumed insects (*n* = 69, 34.3%). These responses indicated that awareness and experience of entomophagy were slightly higher (by ~10%) than reported in a previous study investigating Australian consumer awareness and acceptance of insects as food [17]. This may reflect industry growth and/or increased exposure to edible insects (e.g., via coverage in popular media) in recent years, both in Australia and across the world.

Participants were segmented into different groupings based on their: (i) food neophobia, with ‘neophiles’ (*n* = 92) and ‘non-neophiles’ (*n* = 109) identified; (ii) prior insect consumption, i.e., ‘insect-eating consumers’ (*n* = 69) and ‘non-insect-eating consumers’ (*n* = 132); and (iii) dog-human relationship, with three cluster groups identified, being ‘dog people’ (*n* = 56), ‘dog parents’ (*n* = 99), and ‘dog owners’ (*n* = 46). The ‘dog people’ cluster had the highest mean scores for questions related to dog-oriented self-concept and anthropomorphism (Table 2). Thus, ‘dog people’ were most likely to ‘humanize’ their dogs and have a relationship interwoven with their own life and identity, similar to the corresponding cluster reported in the study from which the dog-human relationship items were adopted [48]. ‘Dog parents’ considered their dogs to be part of their family, but they were less likely to see their dogs playing an integral role in their personal and social identities than ‘dog people.’ While ‘dog owners’ also considered their dogs to be part of the family, participants in this cluster generally responded with the lowest scores (Table 2), indicating they were the least likely to ‘humanize’ their dog, which is, again, similar to the ‘pet owner’ cluster reported in the aforementioned study [48].

### 3.2. Dog Food Choices and Factors Influencing Purchasing Decisions

Participant responses indicated dog food was typically purchased from pet stores (53.2%) and supermarkets (53.2%) (Table 3), which was consistent with findings for cat and dog owners in other studies [47,48]. Only 22.9% of participants purchased pet food online, 17.4% from veterinary clinics, and 13.9% from butchers. Most participants fed their dogs a diet comprising ‘a combination of dry and canned/wet food’ (31.8%), ‘primarily dry food’ (26.4%), or a ‘combination of dry and home-prepared food’ (23.9%). Previous studies have similarly found dry food to be popular amongst dog owners [47,53], with the latter study finding ~86% of dogs’ diets comprised dry food [53]. Finally, a substantial proportion of participants indicated that they fed their dogs treats at least twice a week (77.1%), with ~45% feeding their dogs treats daily. These results suggest that dry dog food and/or treats might be suitable options for supplementation with insect meal.

When purchasing dog food, participants placed the greatest importance on health/nutrition (mean = 6.4), followed by palatability (mean = 5.7) and premium quality (mean = 5.5) (Table 4). Trends in pet food determinants support previous research by Boya and colleagues, who found that health/nutrition, quality, freshness, and taste were highly important to consumers when purchasing dog food [46]. A recent study by Schleicher and colleagues [53] also reported nutrition as the highest motivator of dog food purchasing decisions. In the current study, ‘recommendations from a veterinarian’ were the most influential information source when choosing dog food (mean = 5.4). In support of this finding, several studies have found dog food choice to be highly influenced by veterinarian approval [53,54,55], including for insect-based pet food [32]. Interestingly, in the current study, participant responses indicated that the price of dog food was only of moderate importance (mean = 4.7). Again, this was in agreement with previous research [46,53]. Nevertheless, price is an underlying factor in consumer choice and purchasing behavior since price affects consumers’ willingness and ability to pay for goods and services [56]. Insect protein is still relatively expensive compared to other protein sources, largely due to the small scale of most insect rearing operations. Insect-based pet foods might, therefore, be prohibitively expensive for some dog owners, despite survey responses suggesting that price was less important than factors such as health/nutrition, palatability, and quality (Table 4). As production expands and economies of scale are realized, insect protein, and therefore insect-based pet foods, can reasonably be expected to become more affordable.

When responses from the different participant segments were considered, significant differences were observed between the three dog-relationship clusters (Table 4), whereas no significant differences were found between consumers segmented by food neophobia or previous insect consumption (Table S1). ‘Dog people’ tended to have higher mean scores for pet food choice determinants compared to ‘dog parents’ and ‘pet owners’ (with the exception of ‘brand name’, ‘being on sale’, ‘product selection in store’, and ‘convenience of store location’, Table 4), supporting the previous findings of Boya and colleagues [46]. ‘Dog people’ had statistically higher mean scores than both ‘dog parents’ and ‘pet owners’ for health/nutrition, premium quality, and environmental sustainability. Regardless, all dog-relationship segments followed the trends observed for the total sample, i.e., palatability, health/nutrition, and premium quality were rated the highest for each group. Participant responses indicated the health/nutrition of their dogs’ food (mean = 6.4) was of greater importance than their own food (mean = 6.0; Tables S2 and S3), further emphasizing that health/nutrition is a highly influential factor in choosing dog food. This was also observed for each of the dog-relationship clusters (Table S4).

### 3.3. Importance of Environmental Factors for Food Choice

Most participants were not highly concerned about the environmental impacts of their own diet or that of their dogs (Table 5). Around 40% of participants indicated that they had changed their personal diet for environmental reasons, while 26% were planning to alter their diet within the next 12 months for environmental reasons. Most owners did not consider the environmental impact of their dogs’ diet (62.2%), and, consequently, only 10% of owners had changed their dogs’ diet for this reason. Neophiles were slightly more environmentally conscious than non-neophiles (i.e., ≤6.4% difference in scores for all questions). Following a similar trend to food neophobia segments, previous insect consumers were slightly more driven by environmental factors than non-insect consumers (i.e., ≤10.8% difference in scores for all questions). ‘Dog people’ were slightly more environmentally conscious than ‘dog parents’ (i.e., ≤11.6% differences in scores for all questions) but were considerably more environmentally conscious than ‘dog owners’ (i.e., ≤28.3% differences in scores for all questions). These findings support the idea that ‘dog people’ place more importance on environmental sustainability when making pet food purchasing decisions (Table 4). Regardless, all segment responses indicate only moderate concern over the impact of their own food and their dogs’ food on the environment. However, with concerns over climate change increasing in recent decades, people may become more aware of sustainable food choices and seek to alter their personal and/or dogs’ diets. Recent studies suggest that awareness of the environmental benefits of insects increases acceptance of and willingness to purchase insect-based products [28,30]. The environmental sustainability of insect protein could, therefore, be used to help drive the market appeal of insect-based pet foods.

### 3.4. Dog Owners’ Willingness to Feed Their Dogs Insect-Based Dog Food

Table 6 reports the willingness of participants to feed their dogs pet food made with crickets, mealworms, or BSFL. Mealworms and crickets were rated equally, with participants being reasonably positive towards pet food made with these insects (mean = 5.2). BSFL had a significantly lower score but was still rated positively (mean = 4.6). Participants may have perceived mealworms and crickets to be a more suitable option due to greater awareness of these insect species being farmed for human consumption. Additionally, participants may have a sense of disgust towards BSFL being utilized in pet food, as flies are often seen as a nuisance species or carriers of disease and are commonly associated with decomposition [2,57]. It is well recognized in the literature that disgust creates hesitance towards eating insects [7,15,19] and has also been reported in relation to the consumption of livestock reared on insects [23]. Thus, a slightly positive score for BSFL is promising. Overall, participants appear to be reasonably willing to feed their dogs insect-based pet foods.

Higa and colleagues explored US pet owner acceptance of insect-based pet food and reported participants were willing to feed their dogs crickets, BSFL, mealworms, and ants in various forms, i.e., as ‘dog treats with 20% insect flour’, ‘dog food with 20% insect flour’, or ‘a bowl of dried insects’ [26]. In contrast to the current study, participants gave relatively neutral responses (~50 out of a possible 100) for all of the insect species incorporated (as a flour/meal) in dog food or treats, and BSFL was favored over mealworms [26]. BSFL preference may have been higher in this study as, prior to a focus on insect use in dog food, participants were asked to rate acceptance of BSFL for human consumption (e.g., BSFL flour in cookies) and feeding BSFL to livestock and fish, which may have influenced perceptions of BSFL use as food or feed. The decreased willingness of US pet owners to feed their dogs insects (compared with participants in the present study) could reflect cultural differences. Higa and colleagues also found participants were less willing to feed their dogs whole insects than pet food made from insect flour [26], while a more recent study reported capsule, powder/flour, and sachet/snack formats were consumers’ preferred approach to incorporating insects into their pets’ diets [29]. These findings are not surprising, as consumer studies of insect-based foods for humans have found stronger preferences for the consumption of insects incorporated into familiar foods rather than whole insects [16,19]. As mentioned above, dry dog food is typically the most popular food choice [47,53], suggesting processed dry dog food supplemented with insect meal could be received favorably. Also, considering treats were fed almost daily to dogs by many of the participants (45.3%), they may also be a preferable insect-based product type. Interestingly, most participants (70.3%) were more willing to feed insects to their dogs than to eat insects themselves. This agreed with the literature suggesting that people are generally more willing to consume livestock reared on insects than to consume insects directly [21,24,58].

Plant-based products were found to be significantly less acceptable than insect-based products and received neutral scores (Table 5), both overall (mean = 4.0) and by the various consumer segments (range = 3.9–4.1). Participants may have been concerned about the nutritional quality of plant-based pet food, given that health/nutrition was an important factor influencing pet food purchasing decisions (mean = 6.4) (Table 4). Dodd and colleagues reported dog owners were worried about the nutritional completeness of plant-based diets [59]. Concerns over the nutritional quality and potential health risks of these diets, especially over the long term, have certainly been raised previously [36,60].

Participants were also given the opportunity to provide comments on issues or concerns they had with insect-based pet foods, and 126 participants responded. Concerns referenced health/nutrition (58.7%, *n* = 75) and palatability (25.4%, *n* = 32) of insect-based pet foods (Table S5), further reinforcing that these were important dog food choice factors for participants. Several participants also mentioned concerns over food safety (7.9%, *n* = 10) and cost (1.6%, *n* = 2). Previous studies have suggested that acceptance of entomophagy can be influenced by education on the health/nutritional benefits, enjoyable consumption experiences, assurance of food safety, and competitive price points [19,28,29,30,61,62,63]. These results provide insight that pet food manufacturers should consider when making and marketing insect-based pet foods.

Neophiles were more accepting of insect-based pet food than non-neophiles, with significantly different responses for crickets (*p* < 0.001), mealworms *(p* < 0.001), and BSFL *(p =* 0.002). People with lower food neophobia have been shown to demonstrate more positive attitudes towards insect consumption [15,17,64] and the consumption of livestock reared on insects [23,24]. Hence, it is reasonable to assume that non-neophiles would be less willing to feed their dogs a novel pet food made with insects, as seen in this study. Results could also reflect neophiles being more highly educated (i.e., 69.6% had a tertiary qualification compared to just 48.6% for non-neophiles; Table 1) and more environmentally conscious with respect to food choices compared to non-neophiles (Table 5).

Insect-eating consumers were generally accepting of each insect species being used for pet food (all scores > 5.0, Table 6), but, notably, this segment was significantly more willing to feed their dogs BSFL-based pet food (mean = 5.1) than non-insect-eating consumers (mean = 4.4). This likely reflects this segment’s prior exposure to edible insects and/or positive insect consumption experiences, which have been shown to increase acceptance of entomophagy [19,62,64]. Interestingly, insect-eating consumers were also less inclined to feed insects to their dogs than to eat insects themselves (mean = 4.7), whereas non-insect-eating consumers preferred to feed their dogs insects (mean = 5.3). A potential explanation for this is that prior insect consumption has been shown to increase the likelihood of consuming insects again [13,14].

No meaningful differences were observed for insect-based pet food preferences amongst human-dog relationship clusters, with all scores being statistically insignificant (*p*-values were >0.05). Previous studies found the closeness of bonds between dogs and humans to be a poor indicator of acceptance of insect-based pet food [26,28]. However, it is still important to consider these segments in relation to marketing insect-based pet food products as they each place different levels of importance on the determinants influencing purchasing decisions (Table 4), unlike the other segments considered in this study (Table S2). As previously discussed, ‘dog owners’ were clearly more conscious of the health/nutrition, palatability, and environmental impact of their dogs’ diets. People with stronger relationships with their dogs might, therefore, present an ideal target market for insect-based pet food. However, as Fantechi and colleagues noted, without communication of the benefits of insect-based pet food, animal empathy could negatively influence purchasing decisions [28]. With increasing numbers of owners forming strong bonds with their dogs [46,50], it is reasonable to expect growth in the demand for premium pet foods, especially products offering nutritional and health benefits (e.g., insect-based pet foods).

### 3.5. Dog Owners’ and Dogs’ Acceptance of Insect-Based Dog Treats

Fifty dogs (comprising breeds from each of the seven major dog groups, Table 7) and their owners (*n* = 42) participated in the acceptance test. Table S6 reports the demographics of the dog owners, 59.5% of whom were female, with all age groups represented. Almost two-thirds of participants had heard of entomophagy (*n* = 27, 64.3%), but less than one-third had previously consumed edible insects (*n* = 13, 31.0%); however, they generally agreed that their experience(s) eating insects had been positive (range = 4–7, mean = 5.2; 7-point Likert scale). One-third of dog owners indicated they had considered the environmental impact of their dog’s/dogs’ diet (*n* = 14, 33.3%), but only 14% (*n* = 6) had modified their dog’s/dogs’ diet for environmental reasons (in agreement with results from the online survey, Table 5). In contrast, 79% (*n* = 33) indicated they would consider doing so, although this would presumably depend on factors such as health/nutrition, palatability, quality (Table S1), and price.

When dogs were offered insect-based treats, the majority (80%, *n* = 40) fully ate the treat (Table 7), although 30% (*n* = 15) showed some hesitation. One dog partially ate the treat (with some hesitation), while nine dogs smelled but did not eat the treat (18%). Of the 16 dogs identified as being fussy eaters by their owners, 13 fully ate the insect-based treat. Some smaller dog breeds (e.g., the Bishan Frise and a Chihuahua) only ate the treat when their owner broke it into smaller pieces. Conversely, some dogs that did not eat the treat were clearly distracted by the presence of other dogs, but they might have done so in other (quieter) settings. Nevertheless, these results demonstrate that some dog owners are willing to feed their dogs insect-based treats, and importantly, more often than not, dogs, even fussy dogs, were willing to eat them. This finding was in agreement with previous studies that reported the acceptance, safety, and/or digestibility of diets (for dogs) that comprised insect meal or oils [25,26,43]. Given most dog owners in the current study not only fed their dogs a diet that included dry food (70%), but also treats, either daily (78%) or once or twice a week (18%) (Table 7), dry dog food and/or treats would again seem to be appropriate options for supplementation with insect meal.

No attempt was made to formally record the number of dog owners who were invited to participate in the acceptance trial but who declined, nor their reasons for not participating. However, non-participants generally indicated they either didn’t have time or weren’t interested, rather than intimating any concerns over feeding their dog(s) insect-based treats. It would, nevertheless, be reasonable to expect that feelings of disgust and/or concerns around palatability, food safety, quality, and nutritional value would influence some dog owners’ acceptance of insect-based pet food, analogous to findings from the online survey and previous studies related to the use of insects in food [7,13,14,15,16,17].

Given the small sample size (*n* = 50), the effects of dog breed, size, and age on acceptance rates were not considered in the current study. Future research with a larger sample, using a controlled feeding environment, would enable this and overcome the potential for distraction experienced in a public setting.

## 4. Conclusions

Survey participants tended to be accepting of insect-based dog food, with the use of crickets and mealworms in dog food rated more favorably than BSFL, which, in turn, was rated more favorably than plant-based dog food. Neophiles and insect-eating consumers were generally more willing to feed insects to their dogs than non-neophiles and non-insect-eating consumers. Given that the nutrition/health of dogs’ diets was found to be of high importance to survey participants, the nutritional benefits of insect-based dog food could be highlighted on packaging to enhance market appeal. The environmental benefits associated with insect-based protein might also resonate with some consumers. In the subsequent acceptance trial, the majority of dogs that were offered insect-based treats ate them, including a significant proportion of dogs considered to be fussy eaters. The insect-based treats were, therefore, considered to be acceptable to most dogs. Despite a moderate sample size (*n* = 201, whereas 222 survey participants were required for ANOVA with f = 021, a = 0.05, and Power-0.8, for three groups) and limitations associated with a sample bias towards younger, well-educated, and female survey participants, these research findings improve the current understanding of dog owners’ attitudes towards insect-based pet food. Future research could more deeply explore consumer motivations and concerns with feeding insect-based pet food to their dogs. This includes concerns raised by survey participants regarding health/nutrition, food safety, and palatability, as well as the influence of feelings of disgust and/or cost. Insect-based pet foods are, nevertheless, gaining traction in the Australian pet food market and may offer an acceptable compromise until persistent attitudinal barriers to the human consumption of insects can be overcome.

## Figures and Tables

**Table 1:** Demographics, protein consumption, and awareness and consumption of edible insects of online survey participants. Data presented as percentages.

	Total Sample (*n* = 201)	Neophiles (*n* = 92)	Non- Neophiles (*n* = 109)	Insect- Eating Consumers (*n* = 69)	Non-Insect- Eating Consumers (*n* = 132)	Dog People (*n* = 56)	Dog Parents (*n* = 99)	Dog Owners (*n* = 46)
**Sex** ^1^								
Female	82.1	79.3	84.4	82.6	81.8	83.9	82.8	78.3
Male	16.9	20.7	13.8	17.4	16.7	16.1	16.2	19.6
**Age (years)**								
18–24	29.4	30.4	28.4	26.1	31.1	37.5	24.2	30.4
25–34	18.4	16.3	20.2	13.0	21.2	23.2	19.2	10.9
35–44	22.4	23.9	21.1	24.6	21.2	21.4	24.2	19.6
45–54	11.4	13.0	10.1	15.9	9.1	7.1	13.1	13.0
55–64	11.4	8.7	13.8	14.5	9.8	7.1	11.1	17.4
≥65	7.0	7.6	6.4	5.8	7.6	3.6	8.1	8.7
**Education**								
Secondary school	25.4	18.5	31.2	18.8	28.8	21.4	29.3	21.7
Technical/trade certificate	16.4	12.0	20.2	15.9	16.7	19.6	17.2	10.9
Undergraduate university	33.3	42.4	25.7	37.7	31.1	37.5	31.3	32.6
Postgraduate university	24.9	27.2	22.9	27.5	23.5	21.4	22.2	34.8
**Household income (AUD)** ^2^								
<50,000	15.4	14.1	16.5	10.1	18.2	21.4	16.2	6.5
50,000–100,000	32.8	35.9	30.3	30.4	34.1	41.1	28.3	32.6
100,001–150,000	19.9	21.7	18.3	21.7	18.9	17.9	18.2	26.1
150,001–200,000	12.9	14.1	11.9	13.0	12.9	10.7	15.2	10.9
>200,000	7.0	5.4	8.3	7.2	6.8	1.8	8.1	2.5
Prefer not to say	11.9	8.7	14.7	17.4	9.1	7.1	14.1	13.0
**Employment**								
Veterinarian	1.5	3.3	0.0	0.0	2.3	0.0	2.0	1.5
Veterinary technician	3.0	1.1	4.6	1.4	3.8	3.6	3.0	3.0
Animal trainer	3.0	0.0	5.5	2.9	3.0	7.1	2.0	3.0
Pet store employee	2.5	4.3	0.9	1.4	3.0	1.8	4.0	2.5
None of the above	90.0	91.3	89.0	94.2	87.9	87.5	88.9	90.0
**Protein consumption** ^3^								
Red meat	70.1	83.7	58.7	75.4	67.4	62.5	67.7	84.8
White meat	78.1	88.0	69.7	81.2	76.5	67.9	77.8	91.3
Wild meat	10.0	14.1	6.4	17.4	6.1	7.1	12.1	8.7
Fish	67.2	82.6	54.1	78.3	61.4	62.5	69.7	67.4
Seafood/shellfish	29.4	43.5	17.4	40.6	23.5	21.4	29.3	39.1
Eggs	82.1	90.2	75.2	87.0	79.5	80.4	81.8	84.8
Dairy	90.5	95.7	86.2	94.2	88.6	89.3	90.9	91.3
None of the above	4.5	3.3	5.5	0.0	6.8	9.9	3.0	0.0
**Have you previously heard of entomophagy or edible insects?**								
Yes	80.1	83.7	77.1	89.9	75.0	80.4	77.8	84.8
No	19.9	16.3	22.9	10.1	25.0	19.6	22.2	15.2
**Have you previously consumed edible insects?**								
Yes	34.3	41.3	28.4	100	0.0	32.1	32.3	41.3
No	65.7	58.7	71.6	0.0	100	67.9	67.7	58.7

^1^ 1% of participants (*n* = 2) elected not to disclose their sex. ^2^ 11.9% of participants (*n* = 24) elected not to disclose their household income. ^3^ Participants could select more than one protein source.

**Table 2:** Dog-human relationship clusters determined using anthropomorphism and dog-oriented self-concept items.

	Dog People (*n* = 56)	Dog Parents (*n* = 99)	Dog Owners (*n* = 46)	*p*-Value
**Dog-oriented self-concept items**				
My dog(s) is/are my best friend	6.7 a	5.3 b	3.0 c	<0.001
Spending time with my dog(s) prevents me from spending time with other humans	4.3 a	3.1 b	2.4 b	<0.001
My dog(s) have helped me develop better relationships with other people	6.0 a	4.6 b	3.5 c	<0.001
I would not be willing to establish a relationship with someone who was not willing to accept my dog(s)	6.5 a	5.3 b	4.0 c	<0.001
My dog(s) is/are an extension of myself	6.4 a	4.2 b	2.4 c	<0.001
**Anthropomorphism items**				
I see dogs as more like people than wild animals	6.1 a	4.2 b	2.6 c	<0.001
I feel like I can communicate with my dog(s)	6.7 a	5.3 b	5.2 b	<0.001
My dog(s) is/are a part of my family	6.9 a	6.6 b	6.2 c	<0.001
My dog(s) is/are like a child to me	6.6 a	5.7 b	2.5 c	<0.001
I learn a lot from my dog(s)	6.3 a	5.1 b	4.4 c	<0.001
I have the same responsibilities as a parent when it comes to taking care of my dog(s)	6.6 a	5.6 b	4.1 c	<0.001

Values are means, measured on a 7-point Likert scale (where 1 = strongly disagree, 4 = neither agree nor disagree, and 7 = strongly agree). Different letters within a row indicate statistically significant differences (*p* = 0.05, one-way ANOVA).

**Table 3:** Participants’ dog food purchasing and feeding behavior. Data presented as percentages.

	Total Sample (*n* = 201)
**Where do you regularly purchase dog food?** ^1^	
Pet store	53.2
Supermarket	53.2
Online	22.9
Veterinary clinic	17.4
Butcher	13.9
Other	9.0
**Which of the following best describes your dog’s/dogs’ diet?**	
A combination of dry and canned/wet food	31.8
Primarily dry food	26.4
A combination of dry and home-prepared food	23.9
A combination of canned/wet and home-prepared food	4.5
Primarily home-prepared food	3.5
Primarily canned/wet food	2.5
Other	7.5
**How often do(es) your dog(s) get treats?**	
Everyday	45.3
At least once or twice per week	31.8
<Once per week	14.9
<Once per month	5.5
Never	2.5

^1^ Participants could give multiple responses.

**Table 4:** Importance of food choice factors when participants purchase dog food.

	Total Sample (*n* = 201)	Dog People (*n* = 56)	Dog Parents (*n* = 99)	Dog Owners (*n* = 46)	*p*-Value
Advertising	2.3	2.5 a	2.4 a	1.7 b	0.021
Being on sale/promotion/discount	3.9	3.9	4.0	3.6	0.472
Brand name	3.5	3.6	3.7	2.9	0.071
Convenience of store location	4.5	4.4	4.6	4.7	0.574
Ease of preparation	4.8	4.9	4.7	5.0	0.518
Environmentally sustainability	4.7	5.2 a	4.6 b	4.3 b	0.003
Health/nutrition	6.4	6.8 a	6.3 b	6.2 b	0.001
Holistic/natural/organic	4.1	4.7 a	4.3 a	3.3 b	0.001
Palatability	5.7	6.1 a	5.7 ab	5.1 b	0.001
Premium quality	5.5	6.0 a	5.3 b	5.1 b	0.002
Price	4.7	4.9	4.6	4.8	0.437
Product selection in store where food is purchased	4.0	3.9	4.2	3.6	0.219
Recommendations from a veterinarian	5.4	5.7	5.2	5.2	0.160
Recommendations from friends	3.9	4.3 a	3.9 ab	3.3 b	0.021
Recommendations from pet food manufacturers	3.1	3.6 a	3.1 ab	2.4 b	0.001
Social media	1.9	2.1	2.0	1.5	0.055
Type of store where food is purchased	3.5	3.9	3.4	3.2	0.104
Variety in diet	4.7	5.1 a	4.7 ab	4.2 b	0.012

Values are means, measured on a 7-point Likert scale (where 1 = strongly disagree, 4 = neither agree nor disagree, and 7 = strongly agree). Different letters within a row indicate statistically significant differences amongst dog-human relationship consumer segments (*p* = 0.05, one-way ANOVA).

**Table 5:** Influence of environmental concerns on participants’ diet and their dogs’ diets. Data presented as percentages.

	Total Sample (*n* = 201)	Neophiles (*n* = 92)	Non- Neophiles (*n* = 109)	Insect- Eating Consumers (*n* = 69)	Non-Insect- Eating Consumers (*n* = 132)	Dog People (*n* = 56)	Dog Parents (*n* = 99)	Dog Owners (*n* = 46)
**Have you changed your personal diet for environmental reasons?**								
Yes	39.8	42.4	37.6	46.4	36.4	48.2	37.4	39.8
No	60.2	57.6	62.4	53.6	63.6	51.8	62.6	60.2
**Within the next 12 months, are you planning on changing your personal diet for environmental reasons?**								
Yes	25.9	27.5	24.8	27.5	25.2	32.1	23.5	26.0
No	73.6	72.5	75.2	72.5	74.8	67.9	76.5	74.0
**Have you previously considered the environmental impact of your dog’s/dogs’ diet?**								
Yes	37.8	41.3	34.9	44.9	34.1	50.0	38.4	21.7
No	62.2	58.7	65.1	55.1	65.9	50.0	61.6	78.3
**Have you changed your dog’s/dogs’ diet for environmental reasons?**								
Yes	10.0	9.8	10.1	14.5	7.6	16.1	9.1	4.3
No	90.0	90.2	89.9	85.5	92.4	83.9	90.9	95.7

**Table 6:** Participants’ willingness to feed insect- and plant-based pet food products to their dogs.

	Total Sample (*n* = 201)	Neophiles (*n* = 92)	Non- Neophiles (*n* = 109)	*p*-Value ^1^	Insect- Eating Consumers (*n* = 69)	Non-Insect- Eating Consumers (*n* = 132)	*p*-Value ^1^	Dog People (*n* = 56)	Dog Parents (*n* = 99)	Dog Owners (*n* = 46)	*p*-Value ^2^
**I would be willing to feed my dog pet food made from…**											
Mealworms	5.2 a	5.6 a	4.8 a	<0.001	5.3 a	5.1 a	0.247	5.3 a	5.1 a	5.4 a	0.302
Crickets	5.2 a	5.6 a	4.9 a	<0.001	5.4 a	5.1 a	0.214	5.3 a	5.1 a	5.4 a	0.203
BSFL	4.6 b	5.0 b	4.3 b	0.002	5.1 a	4.4 b	0.006	4.6 b	4.5 b	5.0 b	0.204
Plants	4.0 c	4.0 c	3.9 b	0.747	3.9 b	4.1 b	0.487	4.0 b	4.0 b	3.8 c	0.815
*p*-Value	<0.001	<0.001	<0.001		<0.001	<0.001		<0.001	<0.001	<0.001	
**I would be more willing to feed my dog(s) an insect-based pet food than to eat insects or insect-based foods myself**											
	5.1	5.0	5.2	0.242	4.7	5.3	0.008	5.2	5.0	5.2	0.795

Values are means, measured on a 7-point Likert scale (where 1 = strongly disagree, 4 = neither agree nor disagree, and 7 = strongly agree). Different letters within a column indicate statistically significant differences amongst responses within consumer segments (*p* = 0.05, one-way ANOVA), with all *p*-values being <0.001. ^1^ *p*-values from *t*-tests (*p* = 0.05) of responses for participants from corresponding consumer segments. ^2^ *p*-values from one-way ANOVA (*p* = 0.05) of responses for dog-human relationship consumer segments.

**Table 7:** Type, usual diet, and treat frequency of dogs involved in acceptance test their response to insect-based treats. Data presented as percentages.

	Total Sample (*n* = 50)
**Type of dog**	
Working dogs (Siberian Huskie, Schnauzer, Mastiff)	8
Herding dogs (Border Collie, Heeler, Kelpie, Corgi)	22
Hounds (Beagle, Whippet, Greyhound)	10
Sporting dogs (Spaniel, Golden Retriever, Pointer)	20
Non-sporting dogs (Bulldog, Poodle)	14
Toys (Bichon Frise, Cavalier King Charles, Pug, Papillon, Chihuahua)	8
Terriers (Staffordshire, Jack Russell, Yorkshire, Bull)	18
Unknown	2
**Usual diet**	
Primarily dry food	10
Equal amounts of dry and canned/wet food	20
Primarily home-prepared food	18
Equal amounts of dry and home-prepared food	40
Other (kangaroo, biologically appropriate raw food)	12
**Treat frequency**	
Daily	72
Once or twice a week	18
Weekly	4
Monthly	2
Less than monthly	2
Never	2
**Response to insect-based treat**	
Ate fully, without hesitation	50 (14 ^1^)
Ate fully, with some (≤5 s) hesitation	18 (4 ^1^)
Ate fully, with (>5 s) hesitation	12 (8 ^1^)
Ate partially, with some (≤5 s) hesitation	2 (0 ^1^)
Smelled but did not eat	18 (6 ^1^)

^1^ Relative proportion of the dogs (*n* = 16) considered to be fussy eaters by their owners.

## Data Availability

All data are presented in the paper and/or Supplementary Materials.

## Supplementary Materials

The following supporting information can be downloaded at: https://www.mdpi.com/article/10.3390/insects16030290/s1, Table S1: Importance of food choice factors when participants (and segments) purchase dog food; Table S2: Importance of food choice factors when participants purchase their own food; Table S3: Importance of food choice factors when participants purchase their own food compared to their dogs’ food; Table S4: Importance of food choice factors when participants purchase their own food compared to their dogs’ food, by dog-relationship; Table S5: Key concerns raised by participants over feeding their dogs insect-based pet food; Table S6: Demographics, awareness and consumption of edible insects, and concern over environmental impacts of dog’s/dogs’ diets, of acceptance test participants.
