# Supplementary material for: Australian Dog Owners’ Acceptance of Insect-Based Pet Food
Source: Insects. 2025 Mar 11;16(3):290. doi: 10.3390/insects16030290 (PMC11942749; doi:10.3390/insects16030290)
Supplement: Supplementary file 1 [file insects-16-00290-s001.zip › insects-3481220-supplementary.pdf]

insects

MDPI

Supplementary Materials

Article

Australian Dog Owners' Acceptance of Insect-Based Pet Food

Anna Triggs [1], Ishka Bless [1], Lukas Danner [1,2], Maria Saarela [3] and Kerry Wilkinson [1,*]

[1] School of Agriculture, Food and Wine, The University of Adelaide, PMB 1, Glen Osmond, SA 5064, Australia; annatriggs1@gmail.com (A.T.); ishka.bless@adelaide.edu.au (I.B.)
[2] Commonwealth Scientific and Industrial Research Organisation (CSIRO), Agriculture and Food, 671 Sneydes Rd, Werribee, VIC 3030, Australia; lukas.danner@csiro.au (L.D.)
[3] South Australian Research and Development Institute (SARDI), Department of Primary Industries and Regions, GPO Box 397, Adelaide, SA 5001, Australia; maria.saarela@sa.gov.au
* Correspondence: kerry.wilkinson@adelaide.edu.au (K.W.); Tel: +61-8-8313-7360

Table S1. Importance of food choice factors when participants (and segments) purchase dog food.

	**Total Sample (n = 201)**	**Neophiles (n = 92)**	**Non-Neophiles (n = 109)**	***p*-Value [1]**	**Insect-Eating Consumers (n = 69)**	**Non-Insect-Eating Consumers (n = 132)**	***p*-Value [1]**
Advertising	2.3	2.1	2.4	0.077	2.1	2.4	0.241
Being on sale/promotion/discount	3.9	3.9	3.9	0.734	3.9	3.9	0.968
Brand name	3.5	3.3	3.6	0.176	3.3	3.6	0.347
Convenience of store location	4.5	4.5	4.6	0.716	4.7	4.5	0.470
Ease of preparation	4.8	4.8	4.8	0.999	4.9	4.8	0.545
Environmental sustainability	4.7	4.8	4.6	0.237	4.9	4.6	0.136
Health/nutrition	6.4	6.5	6.4	0.242	6.4	6.4	0.975
Holistic/natural/organic	4.1	4.0	4.2	0.355	4.2	4.1	0.816
Palatability	5.7	5.7	5.7	0.908	5.6	5.8	0.384
Premium quality	5.5	5.5	5.4	0.817	5.5	5.4	0.937
Price	4.7	4.8	4.7	0.596	4.7	4.7	0.964
Product selection in store where food is purchased	4.0	3.8	4.2	0.129	4.0	4.0	0.802
Recommendations from a veterinarian	5.4	5.4	5.3	0.454	5.3	5.4	0.647
Recommendations from friends	3.9	3.7	4.1	0.080	3.9	3.9	0.783
Recommendations from food manufacturers	3.1	2.9	3.2	0.350	3.0	3.1	0.544
Social media	1.9	1.7	2.1	0.071	1.8	1.9	0.573
Type of store where food is purchased	3.5	3.4	3.7	0.287	3.5	3.6	0.863
Variety in diet	4.7	4.6	4.7	0.568	4.6	4.7	0.570

Data are presented as means (where 1 = strongly disagree, 4 = neither agree nor disagree and 7 = strongly agree). [1] *p*-values from t-tests (p = 0.05) of responses for participants from corresponding consumer segments.

Table S2. Importance of food choice factors when participants purchase their own food.

	Total Sample (n = 201)	Neophiles (n = 92)	Non-Neophiles (n = 109)	p-Value [1]	Insect-Eating Consumers (n = 69)	Non-Insect-Eating Consumers (n = 132)	p-Value [1]	Dog People (n = 56)	Dog Parents (n = 99)	Dog Owners (n = 46)	p-Value [2]
Advertising	2.5	2.4	2.6	0.338	2.5	2.6	0.842	2.8	2.5	2.2	0.138
Being on sale/promotion/discount	4.6	4.7	4.5	0.397	4.4	4.7	0.197	4.5	4.6	4.7	0.944
Brand name	3.3	3.2	3.3	0.577	3.4	3.2	0.586	3.4	3.3	3.2	0.887
Convenience of store location	5.1	5.3	4.9	0.091	5.0	5.1	0.630	5.0	5.1	5.3	0.641
Ease of preparation	5.0	5.1	4.9	0.257	4.9	5.1	0.450	5.5 a	5.0 a	4.4 b	0.001
Environmental sustainability	5.1	5.2	5.0	0.318	5.2	5.1	0.685	5.6 a	5.0 b	4.7 b	0.003
Health/nutrition	6.0	6.2	5.9	0.038	6.2	6.0	0.136	6.4 a	6.0 ab	5.8 b	0.025
Holistic/natural/organic	4.4	4.4	4.3	0.965	4.4	4.3	0.954	4.5	4.5	3.9	0.135
Palatability	6.2	6.4	6.1	0.118	6.4	6.2	0.058	6.3	6.2	6.2	0.998
Premium quality	5.2	5.4	5.2	0.292	5.3	5.2	0.927	5.6	5.2	4.9	0.057
Price	5.1	5.3	4.9	0.036	4.9	5.2	0.249	5.4	4.9	5.0	0.216
Product selection in store where food is purchased	4.6	4.6	4.6	0.843	4.7	4.6	0.447	4.5	4.7	4.7	0.770
Recommendations from a healthcare professional	4.7	4.9	4.6	0.214	4.6	4.8	0.369	5.5 a	4.5 b	4.3 b	<0.001
Recommendations from friends	4.4	4.6	4.2	0.106	4.2	4.5	0.222	4.5	4.3	4.4	0.686
Recommendations from food manufacturers	2.7	2.6	2.8	0.258	2.6	2.7	0.690	3.0 a	2.7 ab	2.2 b	0.020
Social media	2.1	2.0	2.2	0.287	2.0	2.2	0.576	2.5 a	2.2 a	1.5 b	0.002
Type of store where food is purchased	4.0	4.0	4.1	0.550	4.3	3.9	0.064	4.3	4.0	3.8	0.398
Variety in diet	5.7	6.0	5.5	0.005	6.1	5.5	<0.001	6.0	5.5	5.8	0.055

Values are means, measured on a 7-point Likert scale (where 1 = strongly disagree, 4 = neither agree nor disagree and 7 = strongly agree). [1] p-values from t-tests (p = 0.05) of responses for participants from corresponding consumer segments. [2] p-values from one-way ANOVA (p = 0.05) of responses for dog-human relationship consumer segments; different letters indicate statistically significant differences.

Table S3. Importance of food choice factors when participants purchase their own food compared to their dogs' food.

	**Own Food**	**Dogs' Food**	***p*-Value**
Advertising	2.5	2.3	*0.075*
Being on sale/promotion/discount	4.6	3.9	*<0.001*
Brand name	3.3	3.5	*0.290*
Convenience of store location	5.1	4.5	*<0.001*
Ease of preparation	5.0	4.8	*0.254*
Environmentally sustainability	5.1	4.7	*<0.001*
Health/nutrition	6.0	6.4	*<0.001*
Holistic/natural/organic	4.4	4.1	*0.191*
Palatability	6.2	5.7	*<0.001*
Premium quality	5.2	5.5	*0.143*
Price	5.1	4.7	*0.026*
Product selection in store where food is purchased	4.6	4.0	*<0.001*
Recommendations from a healthcare professional or veterinarian	4.7	5.4	*<0.001*
Recommendations from friends	4.4	3.9	*<0.002*
Recommendations from food or pet food manufacturers	2.7	3.1	*0.026*
Social media	2.1	1.9	*0.160*
Type of store where food is purchased	4.0	3.5	*0.003*
Variety in diet	5.7	4.7	*<0.001*

Values are means, measured on a 7-point Likert scale (where 1 = strongly disagree, 4 = neither agree nor disagree and 7 = strongly agree). *p*-values are from t-tests (p = 0.05).

Table S4. Importance of food choice factors when participants purchase their own food compared to their dogs' food, by dog-relationship.

	**Dog People (*n* = 56)**			**Dog Parents (*n* = 99)**			**Dog Owners (n = 46)**		
	**Own Food**	**Dogs' Food**	***p*-Value**	**Own Food**	**Dogs' Food**	***p*-Value**	**Own Food**	**Dogs' Food**	***p*-Value**
Advertising	2.8	2.5	*0.104*	2.5	2.4	*0.170*	2.2	1.7	*0.011*
Being on sale/promotion/discount	4.5	3.9	*0.010*	4.6	4.0	*<0.001*	4.7	3.6	*0.040*
Brand name	3.4	3.6	*0.391*	3.3	3.7	*0.041*	3.2	2.9	*0.257*
Convenience of store location	5.0	4.4	*0.006*	5.1	4.6	*0.001*	5.3	4.7	*0.001*
Ease of preparation	5.5	4.9	*0.016*	5.0	4.7	*0.021*	4.4	5.0	*0.001*
Environmentally sustainability	5.6	5.2	*0.001*	*5.0*	4.6	*<0.001*	*4.7*	4.3	<0.001
Health/nutrition	6.4	6.8	*0.001*	6.0	6.3	*0.005*	5.8	6.2	*0.012*
Holistic/natural/organic	4.5	4.7	*0.431*	4.5	4.3	*0.044*	3.9	3.3	*0.020*
Palatability	6.3	6.1	*0.458*	6.2	5.7	*<0.001*	6.2	5.1	*<0.001*
Premium quality	5.6	6.0	*0.054*	5.2	5.3	*0.574*	4.9	5.1	*0.363*
Price	5.4	4.9	*0.019*	4.9	4.6	*0.026*	5.0	4.8	*0.154*
Product selection in store where food is purchased	4.5	3.9	*0.066*	4.7	4.2	*0.010*	4.7	3.6	*0.011*
Recommendations from a veterinarian	5.5	5.7	*0.406*	4.5	5.2	*<0.001*	4.3	5.2	*0.001*
Recommendations from friends	4.5	4.3	*0.222*	4.3	3.9	*0.015*	4.4	3.3	*0.001*
Recommendations from food or pet food manufacturers	3.0	3.6	*0.013*	2.7	3.1	*0.013*	2.2	2.4	*0.394*
Social media	2.5	2.1	*0.097*	2.2	2.0	*0.046*	1.5	1.5	*0.900*
Type of store where food is purchased	4.3	3.9	*0.178*	4.0	3.4	*0.007*	3.8	3.2	*<0.001*
Variety in diet	6.0	5.1	*<0.001*	5.5	4.7	*<0.001*	5.8	4.2	*<0.001*

Values are means, measured on a 7-point Likert scale (where 1 = strongly disagree, 4 = neither agree nor disagree and 7 = strongly agree). *p*-values from t-tests ($p = 0.05$) of responses for participants from corresponding consumer segments.

Table S5. Key concerns raised by participants over feeding their dogs insect-based pet food. Data presented as percentages (and no. of participants).

	**Response Rate (*n* = 126)**
Health/nutrition	59.5 (*n* = 75)
Palatability	25.4 (*n* = 32)
Safety	7.9 (*n* = 10)
Digestion	15.1 (*n* = 19)
Price	1.6 (*n* = 2)
Disgust	1.6 (*n* = 2)
Veterinary approval	1.6 (*n* = 2)
Ease of preparation	0.8 (*n* = 1)

Table S6. Demographics, awareness and consumption of edible insects, and concern over environmental impacts of dog's/dogs' diets, of acceptance test participants. Data presented as percentages.

	Total Sample (n = 42)
**Sex**	
Female	59.5
Male	38.1
Non-binary	2.4
**Age (years)**	
18–29	16.7
30–39	26.2
40–49	16.7
50–59	21.4
≥60	19.0
**Have you previously heard of entomophagy or edible insects?**	
Yes	64.3
No	35.7
**Have you previously consumed edible insects?**	
Yes	31.0
No	69.0
**Have you considered the environmental impact of your dog's/dogs' diet?**	
Yes	33.3
No	67.3
**Have you modified your dog's/dogs' diet for environmental reasons?**	
Yes	14.3
No	85.7
**Would you consider modifying your dog's/dogs' diet for environmental reasons?**	
Yes	21.4
No	78.6
